# Gestational Metabolic Risk: A Narrative Review of Pregnancy-Related Complications and of the Effectiveness of Dietary, Exercise and Lifestyle Interventions during Pregnancy on Reducing Gestational Weight Gain and Preventing Gestational Diabetes Mellitus

**DOI:** 10.3390/jcm13123462

**Published:** 2024-06-13

**Authors:** Georgios I. Tsironikos, George E. Zakynthinos, Athina Tatsioni, Vasiliki Tsolaki, Iraklis-Georgios Kagias, Petros Potamianos, Alexandra Bargiota

**Affiliations:** 1 Department of Medicine, University of Ioannina, University Campus, 45110 Ioannina, Greece; g.tsironikos@uoi.gr; 2 3rd Department of Cardiology, “Sotiria” Chest Diseases Hospital, Medical School, National and Kapodistrian University of Athens, 11527 Athens, Greece; 3 Department of Research for General Medicine and Primary Health Care, Faculty of Medicine, University of Ioannina, University Campus, 45110 Ioannina, Greece; atatsion@uoi.gr; 4 Department of Critical Care, University Hospital of Larissa, Faculty of Medicine, University of Thessaly, Mezourlo, 41335 Larissa, Greece; vasotsolaki@yahoo.com; 5 Department of Neurosurgery, University Hospitals Sussex NHS Foundation Trust, Brighton BN2 5BE, UK; i.kagias@nhs.net; 6 Department of Gastroenterology, University Hospital of Larissa, Faculty of Medicine, University of Thessaly, Mezourlo, 41335 Larissa, Greece; petpot92@gmail.com; 7 Department of Internal Medicine-Endocrinology, University Hospital of Larissa, Faculty of Medicine, University of Thessaly, Mezourlo, 41335 Larissa, Greece; abargio@med.uth.gr

**Keywords:** diet, nutrition, exercise, PA, obesity, GWG, GDM

## Abstract

**Objective:** This study is a Narrative Review that aims at investigating the implications of obesity, excessive gestational weight gain (GWG) and gestational diabetes mellitus (GDM). Additionally, this Review seeks to explore the effectiveness of nutrition, and/or exercise interventions during pregnancy on reducing GWG and preventing GDM. **Materials and Methods:** The search in literature included studies that identified obesity, GWG, GDM and associated risks during pregnancy. Also, SR and MA focusing on interventions including diet, or physical activity (PA), or combined (i.e., lifestyle interventions) and their impact on metabolic risk during pregnancy, were identified through searches in PubMed, Cochrane Database of Systematic Reviews (CDSRs), and Scopus. **Results:** The study findings suggest that lifestyle interventions during pregnancy may be effective in reducing excessive GWG. Regarding the prevention of GDM, results from studies evaluating lifestyle interventions vary. However, significant and less controversial results were reported from studies assessing the efficacy of exercise interventions, particularly in high-risk pregnant women. **Conclusions:** Lifestyle interventions during pregnancy may reduce excessive GWG. Exercise during pregnancy may prevent GDM, especially in high-risk pregnant women. Future research is warranted to tailor lifestyle interventions for optimal effectiveness during pregnancy.

## 1. Introduction

The prevalence of female obesity is more pronounced in high-income countries (HICs), with notable rates observed in the United States of America (USA) at 34% and the United Kingdom (UK) at 25% [1]. In HIC, up to 60% of women of reproductive age are either overweight or patients with obesity [2], with approximately two-thirds falling into this category in the USA [3]. The incidence of pre-pregnancy overweight or obesity has nearly doubled over the past three decades [4]. Alarmingly, around half of women are already classified as overweight or patients with obesity at the onset of pregnancy, reaching critical levels [5,6]. This percentage varies globally, with figures such as 60% in the USA, 30% in Europe, and 10% in Asia [7].

Moreover, there is a concerning trend of increasing body mass index (BMI) among women of childbearing age, with an estimated annual gain of about 700 g every five years [8]. Additionally, 25% of women commence pregnancy with preexisting conditions such as hypertension (HY) or hypertriglyceridemia, alongside obesity [9,10]. Despite the challenges during pregnancy, approximately 25% of women continue to be overweight even after childbirth [11].

Gestational diabetes mellitus (GDM) stands out as the most common health issue during pregnancy and childbirth [12,13]. The incidence of GDM is on the rise worldwide, irrespective of the diagnostic criteria applied [2,14]. Following the criteria established by the International Association of Diabetes and Pregnancy Study Groups (IADPSGs), 14.7% of pregnancies globally are complicated by GDM [15]. The prevalence of GDM worldwide, as reported by the International Diabetes Federation (IDF), reached 16.7% in 2021 [16]. In the USA, the prevalence of GDM has plateaued since 2017, following a continuous and nearly doubled increase from 2006 to 2016 [17]. However, in Europe, the prevalence of GDM may approach 24% [13].

Diet plays a crucial role in the management of GDM [13]. However, determining the optimal diet for treating or preventing GDM remains a topic of debate [13]. The preventive impact of lifestyle interventions during pregnancy such as dietary modifications, physical activity (PA), or a combination of both, on GDM is not entirely clear [18]. This study seeks to investigate adverse maternal health conditions, including obesity, gestational weight gain (GWG), and GDM, and examining the effectiveness of lifestyle interventions in reducing the risk of gestational metabolic complications in these conditions.

## 2. Materials and Methods

### 2.1. Search Strategy

The effect of dietary, exercise and lifestyle interventions during pregnancy was evaluated with a Narrative Review of Systematic Reviews (SRs) and Meta-Analyses (MAs). This Narrative Review of SR and MA was performed after a systematic search in PubMed, Cochrane Database of Systematic Reviews (CDSRs), and Scopus. The search included items with registration date between 1 January 2020 and 30 November 2023. The search strategy included keywords related to diet, nutrition, exercise, PA, obesity, GWG, and GDM (Table S1). Eligible SR and MA were selected based on title and/or abstract. Moreover, pregnancy-related complications of obesity, gestational weight gain (GWG), and gestational diabetes mellitus (GDM) were identified through a manual search in literature. Finally, the software of EndNote 21.1 was used for this search.

### 2.2. Eligibility Criteria

The PICO (population, intervention, comparator, and outcome) approach was used for selecting eligible studies.

#### 2.2.1. Population

SR and MA in English language, including pregnant women under dietary, exercise or lifestyle interventions that evaluated any identified metabolic risk were accepted. Studies evaluating gestational-associated metabolic risk complications were assessed.

#### 2.2.2. Intervention

Studies assessing any type of active intervention of diet alone, exercise alone, or both during pregnancy were considered as eligible. We accepted the lifestyle as defined by authors, including a combination of diet and exercise.

#### 2.2.3. Comparator

The comparator arm included standard antenatal care.

#### 2.2.4. Outcomes

The outcomes of this study included the incidence of GWG, and/or GDM. Additionally, studies assessing obesity, GWG and GDM pregnancy-related complications were captured.

### 2.3. Data Extraction

The extracted items included the name of first author and studies’ publication year, the characteristics of participant women according to the gestational metabolic risk, the sample size and the type of intervention with details about exercise intervention (i.e., intensity, duration, supervision). Finally, we recorded the significance of each intervention on reducing GWG and/or preventing GDM.

## 3. Results

**Figure 1:**
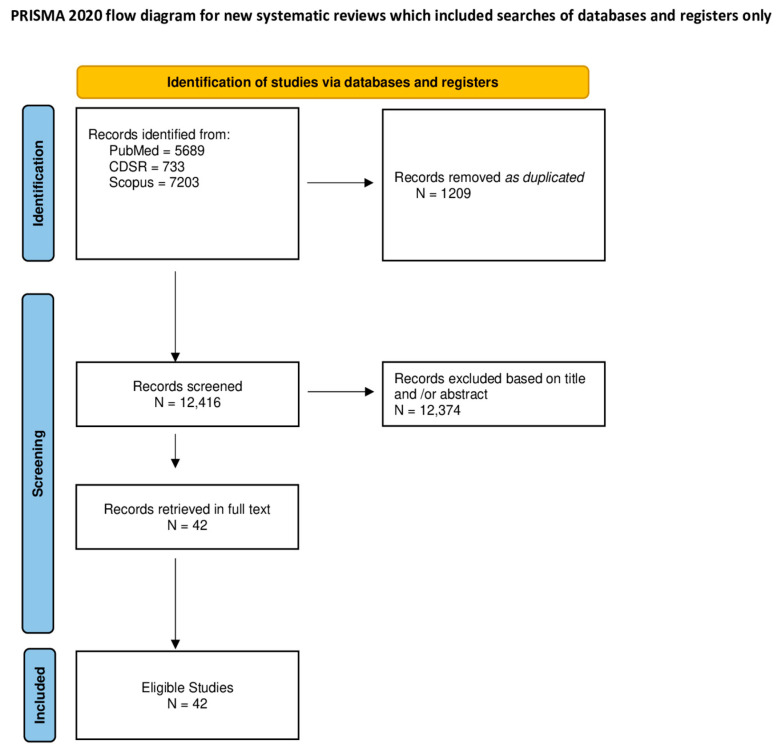
Flow chart of procedures of selecting studies.

A total of 13,625 items were found; 5689 in PubMed, 733 in CDSR, and 7203 in Scopus. After removing 1209 records as duplicates, 12,416 were examined for potential eligibility based on title, or abstract. Among them, 12,374 were excluded. Finally, 42 studies were considered as eligible and included in this study (Figure 1).

### 3.1. Gestational Metabolic Risk and Pregnancy-Related Complications

#### 3.1.1. Obesity

Obesity during pregnancy introduces an increased risk of inflammation [19] and metabolic complications such as insulin resistance and elevated plasma glucose levels [20]. Throughout pregnancy, oxidative stress levels typically rise, accompanied by the activation of antioxidant enzymes to support metabolic processes crucial for placental and fetal development [21]. However, pregnancies characterized by obesity and GDM experience a heightened inflammatory state in addition to the normal metabolic changes in maternal physiology, including increased oxidative stress, insulin resistance, and dyslipidemia [22]. Elevated levels of inflammatory factors may contribute to insulin resistance and the development of GDM [23]. Furthermore, oxidative stress is implicated in the occurrence of GDM [21]. An imbalanced antioxidant system and disturbances in lipid peroxidation status during pregnancy may also play a role in the circumstances leading to GDM [21].

The impact of high maternal weight on pregnancy has been extensively researched and is positively correlated to BMI [5]. Pre-pregnancy overweight and obesity exhibit a robust association with GDM [2,23,24]. An increased pre-pregnancy BMI of 10% has been correlated with a 10% greater risk for both GDM and pre-eclampsia (PE) [20]. Maternal overweight and obesity elevate the risk of GDM by 2.4–3.5 times [25]. Specifically, the risk of GDM in overweight women (BMI 25–30) is rising 6.5-fold, while in patients with obesity, is rising even higher, approximately reaching 17%, compared to 1–3% in normal-weight women [26]. The degree of obesity is directly associated with the risk of GDM, with overweight, obese, and patients with extreme obesity facing two-fold, four-fold, and eight-fold increased risks, respectively [27,28].

Importantly, obesity may lead to adverse maternal and neonatal outcomes, both short-term and long-term, independent of glucose tolerance [2,29]. Furthermore, the combination of overweight or obesity and GDM exacerbates the negative effects during pregnancy, compared to each condition independently [30].

Obesity during pregnancy significantly increases the risk of maternal, fetal, and neonatal morbidity and mortality [20,31]. In the UK, approximately 50% of women who experience mortality during pregnancy or postpartum are overweight or patients with obesity [32]. Apart from GDM, obesity can lead to insulin resistance [20], excessive GWG [20], gestational HY and PE [5], neural tube defects [31], congenital anomalies [31,33], intrauterine fetal death [34], stillbirth [1,19], fetal growth disorders [30], high birth weight or macrosomic infants [5], neonatal hypoglycemia [24], large-for-gestational-age infants [35], shoulder dystocia [36], clotting disorders [26], thromboembolic diseases [31,34], miscarriage [24], pre-term birth (before 37 GW) [33], intrapartum interventions [26], labor induction and cesarean section [5,31], postoperative complications [30], infections [34], wound infections [26], urinary tract infections and endometritis [31], postpartum hemorrhage [34], anemia [31], stress [31], urinary incontinence [31], depression [31], longer hospitalization [4,19], neonatal intensive care unit admission [19], difficulties with breastfeeding [31], and a lower ratio of breastfeeding influencing infant’s growth [19].

Independent of the offspring’s birth weight, maternal obesity can contribute to various adverse health outcomes in infants, children, young adults, and future generations [5,37], including an increased risk of obesity [4,33], the development of Type-2 Diabetes Mellitus (T2DM) [33], metabolic syndrome [4,31], cardiovascular diseases (CVDs) [19], and cardiovascular events, leading to premature mortality in later life [4]. Maternal long-term consequences for pregnant patients with obesity include a heightened lifetime risk of developing T2DM, CVD, depression, orthopedic diseases, and specific types of cancer, including reproductive cancers [38].

#### 3.1.2. Gestational Weight Gain

Irrespective of pre-pregnancy BMI, excessive GWG is associated with increased fat accretion, leading to endothelial dysfunction that elevates the risk of glucose intolerance, GDM, and PE [20]. Therefore, GWG consists of a distinguished GDM risk factor [28]. Both pre-pregnancy BMI and GWG have been identified as two of the most important risk factors for GDM [23] and contribute importantly to the occurrence of large-for-gestational-age infants [3]. Additionally, they increase the risk of gestational HY, fetal growth malformations, premature birth, cesarean delivery, deep vein thrombosis, and wound infections [25]. The combination of maternal obesity and excessive GWG can synergistically increase the incidence of GDM, large-for-gestational-age newborns, postpartum weight retention (PPWR), and childhood obesity [7]. The risk of PPWR doubles after excessive GWG [8]. Particularly, more than 70% of normal-weight mothers who gain excessive weight during pregnancy have been estimated to retain more than five kilograms (kg) one year after postpartum [8].

GWG can independently contribute to the development of adolescent and early adulthood obesity, perpetuating the cycle of female obesity [39]. A birth weight above four kg is well-associated with childhood obesity [8]. Additionally, extreme GWG during the first gestational period is strongly correlated with later-life obesity [37]. Each kg gained during pregnancy is associated with a 1.08-fold increased risk of early childhood obesity [8]. There is also a significant impact on fecundity, with women who have experienced excessive GWG exhibiting lower fertility rates in later years [38].

#### 3.1.3. Gestational Diabetes Mellitus

GDM is a condition in glucose and carbohydrate metabolism that is diagnosed during pregnancy [29]. It is a heterogeneous disorder influenced by genetic, environmental, and physiological risk factors [2,15]. GDM is characterized by impaired pancreatic function of b-cells and insulin resistance, stemming from the physiological, metabolic, and endocrine changes induced by local and placental hormones during pregnancy to meet the growing fetus’s demands for nutrients and oxygen [2,15,27]. The imbalance between insulin secretion and resistance contributes to the development of GDM [27].

GDM has significant complications for children and mothers, both short-term and long-term [27,40]. The adverse effects of GDM have the potential to perpetuate vicious cycles across generations [41]. Fetuses in GDM pregnancies often experience a hyperglycemic state, requiring increased insulin secretion to absorb maternal glucose [42]. The GDM-associated complications exhibit a linear relationship with maternal glucose levels [14]. Consequently, pregnancies that are affected by GDM are at a heightened risk of perinatal morbidity [39,43], and an increased incidence of perinatal mortality [44].

Short-term unfavorable results include gestational HY [40], PE [45] and neonatal hypertensive disorders [23], fetal hypoglycemia [15], neonatal hypoglycemia [19,42], neonatal hypocalcemia [42], congenital malformation [42] and impaired development [13], fetal death [42], polyhydramnios [40], fetal hyperinsulinemia [27], overweight fetus [42], abnormal infant birth weight [13], large-for-gestational-age neonates [44], macrosomia [40,42], shoulder dystocia [46], respiratory distress syndrome [42,46], asphyxia [46], infant hyperbilirubinemia [47] and perinatal jaundice [15,42], increased birth weight [48], birth injuries [15], antepartum and postpartum hemorrhage [49], preterm birth [40], induction of labor [49], cesarean section [40,43], infections [50], and admission to a neonatal care unit [27,48].

Long-term consequences include overweight or obesity, T2DM, metabolic syndrome, CVD, and neuropsychological deficits for both mothers and their offspring [18,40,45]; delayed neurocognitive development [16], and Type-1 Diabetes Mellitus (T1DM) for children [39]; chronic kidney disease [16], cancer [16], psychological burden [49], and depression for mothers [51]. Two or more GDM pregnancies may increase the overall risk of breast cancer (BC), and especially estrogen receptor-positive BC [52]. Additionally, cardiovascular risk is increasing in offspring of mothers with metabolic risk factors in pregnancy, including obesity, HY, and hyperlipidemia [9].

### 3.2. Efficacy of Lifestyle Interventions during Pregnancy on Optimizing Gestational Weight Gain and Preventing Gestational Diabetes Mellitus

#### 3.2.1. Lifestyle Interventions and Gestational Weight Gain

An SR and MA synthesizing any type of lifestyle intervention designed to limit GWG reported a significant reduction in GDM’s incidence with either diet or exercise intervention alone, and no additional benefit with the mixed approach of them [53]. However, a previous MA investigating the effect of lifestyle interventions during pregnancy designed to limit excessive GWG on pregnancy outcomes, did not find a significant association between lower GWG and GDM’s incidence [54] (Table 1, Table 2 and Table 3).

An ΜA exploring the effect of diet, and/or PA in pregnancy found that only dietary intervention was beneficial in reducing significantly GDM [32] (Table 1, Table 2 and Table 3). Additionally, any type of intervention minimized GWG significantly [32] (Table 1, Table 2 and Table 3). Another SR and MA for the USA Preventing Service Task Force (USPSTF) evaluating active lifestyle interventions during pregnancy administrating the Institute of Medicine (IOM) guidelines designed to limit GWG supported that women were less likely to develop GDM [74] (Table 1 and Table 2). An achievement of a healthy GWG was also demonstrated [74]. Women undergoing those specific interventions were less possible to experience PPWR at 12 months [74]. On the contrary, a non-significant result was reported for less weight retention until sixth months after pregnancy [74]. According to a recent SR and MA, any intervention of diet, or PA, or both of them succeeded significantly less GDM and excessive GWG occurrences, as well [77] (Table 1 and Table 2).

On the other hand, a previous MA including randomized controlled trials (RCTs) of dietary and lifestyle interventions attempting to reduce GWG in normal-weight pregnant women advocated a non-significant difference in GDM’s incidence [8] (Table 1 and Table 2); however, the decrease in GWG was significant [8] (Table 1 and Table 2). Moreover, the effect of interventions on PPWR was doubtable [8]. Data regarding the trimester of GDM diagnosis were missing [8]. Three SR and MA assessing lifestyle interventions during pregnancy including diet and PA in overweight and pregnant patients with obesity reported no prevention for GDM, despite the significant differences in lowering excessive GWG [36,45,68] (Table 1, Table 2 and Table 3). Additionally, one of them reported non-significant results for the prevention of GDM [68] (Table 2), and another one reported non-significant results in preventing GDM [45]. In the same line, a multicenter RCT that was conducted in nine European countries revealed no effect for either diet, or PA, or both of them on GDM’s occurrences, whereas GWG was significantly lower in the group of complex intervention [81].

The efficacy of lifestyle interventions on reducing GWG is strengthened by another SR and MA supporting the benefit of either diet, or exercise, or both of them [58] (Table 1, Table 2 and Table 3). A statistical significance was reported for less PPWR by the pool effect of lifestyle interventions [58]; however, less weight retention after pregnancy was non-significant with both diet and counseling intervention, and diet plus supervised intervention [58]. Finally, the International Weight Management in Pregnancy (i-WIP) Collaborative Group performing an MA found significant reduction in maternal GWG either with dietary-only or PA-only interventions or with a mixed approach of them [66] (Table 1, Table 2 and Table 3); however, an effect of lifestyle interventions on preventing GDM was revealed only with PA [66] (Table 2 and Table 3).

#### 3.2.2. Dietary Interventions and Gestational Diabetes Mellitus

Exploring the efficacy of dietary intervention during pregnancy on preventing GDM and ameliorating GWG in the general population, one SR and MA reported significant results for the Mediterranean diet [10] (Table 1 and Table 2). On the other hand, a previous SR and MA did not find significant reduction in GDM by applying any pattern of nutrition intervention [33] (Table 1 and Table 2); however, the reduction in total GWG was significant [33] (Table 2). Similarly, a subsequent SR and MA demonstrated marginally non-significant results [63] (Table 1 and Table 2); however, a protective effect for GDM was feasible with dietary interventions in pregnancies that were complicated by obesity [63].

A recent network MA supported a significant reduced incidence of GDM with probiotic and exercise intervention [78] (Table 1, Table 2 and Table 3). On the contrary, a previous SR and MA [73] (Table 1 and Table 2), and an overview of SR [82] demonstrated that probiotics were not effective in preventing GDM; additionally, they increased the risk of PE and pregnancy hypertensive disorders [73]. Moreover, a network MA evaluating different interventions for GDM’s prevention in overweight or pregnant patients with obesity reported no effect with probiotics [71] (Table 1 and Table 2).

VitD deficiency may be associated with GDM [46,83]. Regarding vitD supplementation, an overview of SR reported a significant effect for GDM prevention [82]. Additionally, an SR and MA evaluating the effect of vitD supplementation on glucose and lipid metabolism in gestational diabetes reported a significant decrease in low- density lipoprotein (LDL)-cholesterol, and improvement in insulin sensitivity; however, it did not reveal an improvement in FPG, hemoglobin A1C (HBA1C), total- and high-density lipoprotein (HDL)-cholesterol, and triglycerides [84]. Furthermore, a network MA found that vitD was not superior compared to the placebo in preventing GDM in pregnant patients with obesity [71] (Table 1 and Table 2).

An SR and MA [16] (Table 1 and Table 2) and an overview of SR [82] reported that supplementation of inositol and myo-inositol may significantly decrease the incidence of GDM, respectively. Contrariwise, a network MA assessing a combination of them with diet and PA intervention did not find any change in GDM’s risk [78] (Table 1 and Table 2).

An SR and MA and an overview of SR investigating omega-3 supplementation in pregnancy resulted in non-significant reduction in metabolic markers (i.e., FPG, insulin resistance, total cholesterol, LDL-cholesterol, triglycerides), and of GDM’s outcome, respectively [22,82]. However, omega-3 increased significantly HDL-cholesterol and reduced C-reactive protein (CRP) [22]. Moreover, two MAs reported no effect on preventing GDM with fish oil supplementation [59,61] (Table 1 and Table 2).

#### 3.2.3. Exercise Interventions and Gestational Diabetes Mellitus

Six SR and MA evaluating RCTs implementing exercise to prevent GDM during pregnancy revealed significant results [44,50,60,64,65,75] (Table 1, Table 2 and Table 3). Furthermore, a significant reduction in GWG was reported in two of them [50,75] (Table 1, Table 2 and Table 3). Moreover, an SR and MA including RCTs and cohort studies that investigated the effect of exercise intervention of high activity compared to low PA initiating before or during early pregnancy, found a protective effect for GDM [85]. Conversely to this trend, a previous SR and MA found no significant difference for the risk of GDM with exercise intervention [55] (Table 1, Table 2 and Table 3).

The results of SR and MA assessing the efficacy of PA on preventing GDM in overweight and high-risk pregnant patients with obesity are controversial. Three SR and MA advocated the preventive role of PA for GDM [19,62,76] (Table 1, Table 2 and Table 3), and especially of aerobic exercise [62,76] (Table 3). Additionally, one of them showed significant reduction in GWG [19] (Table 2 and Table 3). However, no effect for GWG was reported with any type of exercise in the other one [76] (Table 2 and Table 3). On the contrary, a preventive effect for GWG was demonstrated with intervention of metformin [76]. Three SR and MA reported no effect of exercise for GDM [11,20,27] (Table 1, Table 2 and Table 3). Despite non-significant findings for GDM, two of them showed significant lower GWG [11,20] (Table 2 and Table 3). Neither exercise intervention, nor interventions of probiotics, vitD, and metformin managed to reduce GDM, according to a network MA [71] (Table 1 and Table 2); however, PA and metformin reduced GWG [71] (Table 2 and Table 3).

An MA investigating the effect of exercise during pregnancy in previous sedentary or pregnant women with low PA levels reported significant results for both preventing GDM and minimizing GWG [39] (Table 1, Table 2 and Table 3). Moreover, two MA evaluating exercise during pregnancy in high-risk pregnant women, revealed significant results for GDM’s prevention [72,79]; one of them with initiation of exercise before the 20 GW [72], and the subsequent independent to the starting time period of PA [79] (Table 1, Table 2 and Table 3).

#### 3.2.4. Lifestyle Interventions and Gestational Diabetes Mellitus

Regarding studies aiming at preventing GDM, there are also varied reports. An MA reported that either diet, or exercise interventions during pregnancy could prevent GDM [18]. Another MA evaluating the effectiveness of exercise or exercise plus diet interventions during pregnancy in preventing GDM, revealed a beneficial effect for exercise-only interventions [70]. Additionally, according to a recent network MA, GDM could be prevented with the implementation of exercise plus probiotic intervention, whereas dietary only, or dietary plus PA interventions did not alter the incidence of GDM [78] (Table 1, Table 2 and Table 3).

On the contrary, a previous MA reported no differences for GDM’s outcome applying both dietary-based, and dietary plus lifestyle interventions [56] (Table 1 and Table 2). In the same line, a following overview of SR did not demonstrate benefit with diet, or exercise, or a combination of them in preventing GDM [82]. Investigating the effectiveness of diet or PA or both combined interventions for reducing gestational diabetes, an MA revealed a preventive role of separate diet or PA approach and non-significant results for the mixed intervention [69] (Table 1, Table 2 and Table 3). Additionally, two previous SR and MA identifying mixed nutrition and PA interventions did not recognize any beneficial action on GDM’s prevention [57,67] (Table 1 and Table 2). One of them reported non-significant results for restricting GWG [57] (Table 2). Regarding long-term health outcomes, there was no effect for less weight retention after delivery with combined lifestyle interventions during pregnancy for the general population [57]. Considering BMI, significant results were reported for less PPWR for normal-weight women [57]. However, the data for time period of screening and GDM diagnosis were insufficient [57]. Thus, a possible correlation between the trimester of GDM diagnosis and further complications was not feasible. The other SR and MA reported significant less GWG [67]. Additionally, a protective effect on postpartum weight-retention was also demonstrated [67]. GDM’s diagnosis, when reported, was set during the third trimester of pregnancy [67]. Moreover, there was no benefit of the mixed intervention in childhood obesity [67]. Notably, for this long-term consequence, the data for the diagnosis of GDM concerned the last trimester of pregnancy [67].

Finally, according to a recently published SR and MA, exercise intervention during pregnancy may be superior to dietary interventions in preventing GDM in high-risk women [80]. However, the Mediterranean diet may be effective in preventing GDM [80] (Table 1, Table 2 and Table 3).

## 4. Discussion

The findings of this study underscore the urgent need for effective interventions to address the rising prevalence of obesity among women of reproductive age, especially in HIC, and highlight the potential health risks associated with pre-pregnancy overweight and obesity. Obesity’s long-term health implications emphasize the importance of addressing maternal obesity not only for the immediate health of the mother and child, but also for the prevention of chronic diseases and associated risks throughout the lifespan. Physical exercise programs have a certain structure including duration, repetition and intensity. They also give the possibility of supervision. Thus, their effectiveness in preventing GDM and/or lowering GWG compared to dietary or dietary plus physical exercise without the presence of an expertise may differ. The results of studies assessing the effect of exercise interventions attempting to minimize GWG and/or preventing GDM may vary due to differences in the intensity of physical exercise programs, the duration (i.e., initiation, repetition, minutes per session) and supervision. Additionally, the sample size may contribute to the effectiveness of exercise interventions. Comprehensive strategies aimed at managing maternal weight before, during, and after pregnancy are crucial to break the intergenerational cycle of obesity and its related health complications. The findings regarding GWG highlight the long-reaching consequences of excessive GWG, emphasizing the importance of appropriate weight management during pregnancy for the health of both the mother and child across the life course. Maintaining optimal blood glucose levels through dietary, exercise or both interventions during pregnancy, the well-being of both mothers and fetuses is ensured. The increasing prevalence of GDM globally is significant. GDM consists of a major health concern during pregnancy necessitating ongoing attention and effective preventive strategies. Preventing GDM is crucial not only for the immediate health outcomes of the mother and child, but also for breaking potential intergenerational cycles of complications associated with this condition.

### 4.1. Obesity

Overweight and obesity pose significant challenges to global public health, contributing to increased morbidity and mortality [8,76]. In addition to GDM, the prevalence of obesity and DM is also on the rise [86]. Over the past few decades, there has been a substantial increase in the incidence of overweight and obesity [62]. Currently, obesity is considered epidemic [72] and is the most common medical condition worldwide [77]. In 2013, the prevalence of overweight and obesity reached 38% in women and 36.9% in men globally, compared to 29.8% in women and 28.8% in men in 1980 [19]. Currently, approximately 40% of adults are classified as overweight or patients with obesity [87].

The Academy of Nutrition and Dietetics has articulated its position on addressing female obesity in reproductive age through comprehensive and sustained interventions [38]. The recommended approach includes behavioral counseling for diet and PA across various stages: preconception, during pregnancy, and postpartum with a suggested duration of at least 12 to 18 months [38]. Furthermore, the Academy emphasizes the importance of lifestyle interventions aimed at moderating GWG during pregnancy [38]. Additionally, there is a focus on reducing weight retention postpartum [38].

### 4.2. Gestational Weight Gain

GWG is the calculated difference of weight after delivery and in the beginning of pregnancy [74]. Extreme GWG is epidemic globally [7]. The percentage of a larger GWG than suggested in the USA and in Europe is 20–40% [32]. Excessive GWG is more common in patients with obesity [35], and may affect up to 64% of overweight or pregnant patients with obesity [88]. Adverse effects of GWG in mothers and their offspring may be short-term and long-term [29], similar to maternal obesity [35], irrespective to maternal obesity progression [24], even in normal-weight women [33].

The research interest about appropriate interventions to limit excessive GWG is increasing and consists of the priority for many healthcare organizations [66]. According to the IOM 2009 guidelines, diet and PA during pregnancy for weight balance are very important [29]. The IOM recommended an ideal GWG based on pregestational BMI [29]. Particularly, during pregnancy, women with pregestational BMI ≥ 30 should gain from 5.0 to 9.0 kg [33,37,45]; with pregestational BMI 25.0–29.9 from 7 to 11.5 kg [33]; with pregestational BMI 18.5–24.9 from 11.5 to 16.0 kg [33,45]; and with pregestational BMI ≤ 18.5 from 12.5 to 18 kg [45,89]. Despite these guidelines, pregnancy is associated with greater GWG than recommended for most women [39]. Moreover, overweight and pregnant patients with obesity are twice possible to overlook recommendations compared to normal-weight pregnant women [89]. Further investigation is necessary to determine effective measures for improvement of GWG in high-risk overweight and pregnant patients with obesity [68].

### 4.3. Gestational Diabetes Mellitus

The prevention of GDM during pregnancy is considered a priority [80]. GDM is a well-established predictor of future DM [2]. Women with GDM, as well as next generations are at an elevated risk of developing T2DM [23,37]. Particularly, women with GDM may develop T2DM until 70% in later life [90]. T2DM is diagnosed soon after delivery up to 10% of women with previous GDM [2], and its prevalence may approach 38% within the first postpartum year [25]. The percentage of T2DM is increasing up to 50% within five years after delivery [91]; up to 70% in a 10-year duration of follow-up [2]; and up to 60% in the 16th postpartum year [25]. At a 15-year follow-up duration, weight and BMI are significant T2DM risk factors in addition to the history of GDM [91].

The diagnosis of GDM in late pregnancy may increase the risk of childhood obesity [67]. Investigating the correlation between the time period of GDM diagnosis and unfavorable health circumstances in mothers and offspring later in life might be challenging. GDM not only increases the risk of developing T2DM, but also provides an opportunity for diabetes prevention, given the rising prevalence of T2DM among young individuals [92], and a history of GDM being a recognized as a risk factor for T2DM [18]. However, preventing the progression of GDM to T2DM through lifestyle interventions is doubtable [18,45]. Two SR and MA exploring the preventive role of lifestyle interventions after pregnancy in women who had GDM did not report differences in T2DM incidence [91,92]. On the contrary, two SR and MA demonstrated significant findings for T2DM prevention if lifestyle interventions in women with history of GDM initiate within 6 months, and 3 years after postpartum, respectively [93,94]. Finally, an overview of SR that evaluated the effect of lifestyle and pharmacological interventions on preventing T2DM in women with history of GDM, reported effective results [95]. Investigating the effectiveness of mixed diet and PA interventions for reducing gestational diabetes, an MA revealed a preventive role of the mixed diet and PA approach [69]. However, two previous SR and MA identifying also mixed nutrition and PA interventions did not recognize any beneficial action on GDM’s prevention [57,67]. Probably, the implementation of lifestyle interventions tailored to individuals, according to women’s baseline risk for GDM, in early pregnancy, and including motivational arm would be more effective in preventing GDM.

### 4.4. Diet

The nutritional demands during pregnancy are increased, and they are often followed by a higher food intake [33]. Traditional cultural beliefs of “eating for two” may lead to an exceed intake of calories [33]. The lack of awareness of nutritional and blind consumption of supplementation can result in nutritional imbalances and obesity, adding unfavorable gestational risks [11]. Over-nutrition, and under-nutrition during pregnancy increase adverse short-term perinatal, and long-term later-life circumstances [96]. Negative short-term pregnancy outcomes include GDM, PE, impaired or excessive fetal growth, and preterm birth [96]. On the contrary, an appropriate diet during pregnancy, and a limited GWG improve gestational outcomes [96]. Consequently, pregnant women should be advised to keep the appropriate balance between energy intake and expenditure in order to avoid unwishful GWG [33].

International and national clinical practice guidelines for nutrition in pregnancy recommend rich dehydration; according to two of them the amount should be 2–2.5 L of water daily [96]. Most guidelines do not quantify the consumption of fat, carboxylate, and protein [96]. However, one of them supports an uptake of >175 g of fat daily [97]. Moreover, three guidelines recommend additionally 700–1400 mg/week of docosahexaenoic acid (DHA) [96]. Three of the guidelines suggest 175 g of carboxylates daily [96]. Other three guidelines recommend an uptake of 10–71 g of protein daily [96]. Fiber consumption is advocated by two guidelines, particularly between 28 and 35 g daily [96]. The suggested amount of micronutrition’s intake is notably varied [96]. The daily dose of folic acid ranges from 200 mcg to 800 mcg, of iron from ≤30 to 60 mg, of vitB12 from 2.6 mcg to 80 mg per day, of vitD from ≤50 mcg to 1.8 mg per day, of calcium from 5 mcg to 1.3 g, and of iodine from 150 mcg to 1.7 mg when its administration is suggested [96].

### 4.5. Exercise

The interest in the possible beneficial effect of exercise during pregnancy for both mothers and offspring has been grown through the last two decades [39]. Traditional cultural beliefs of reducing PA, or stopping work to prevent obstetric complications have been abandoned [39]. PA during pregnancy has minimal risk [98]. Previous SR and MA demonstrated safety of exercise during pregnancy, concerning miscarriage, perinatal mortality, congenital anomalies, and mother hyperthermia [99,100].

PA has important advantages for pregnant women, without complications [97]. Exercise during pregnancy increases cardiorespiratory fitness, and decreases GWG [101]. Training regularly during pregnancy promotes the expression of the endothelial nitric oxide synthetase (eNOS), nitric oxide (NO), and oxygen metabolism in placenta, probably reducing the likelihood of GDM and HY [102]. PA can improve the quality of life (QUALY) of pregnant women [60]. Two SR and MA reported that both prenatal [103] and postnatal exercise [104] may decrease postpartum depression. Further benefits include reduced preterm labor, fitness, less back pain, better sleep, and reduced anxiety [97]. Offspring complications of the autonomous nervous system development, growth, and weight may be avoided [75].

PA is recommended in pregnancy guidelines [97]. The WHO recommends continuous workout in pregnancy including aerobic exercise (i.e., walking, swimming), and anaerobic exercise (i.e., strength training) [75]. The American College of Obstetricians and Gynecologists (ACOG) emphasizes the importance of restricting excessive GWG [98]. According to the ACOG 2020 guidelines, healthy pregnant women should accumulate moderate to vigorous exercise, preferably aerobic or combined aerobic and anaerobic, at least 30 min daily, 3–4 times weekly [76]. According to the U.S. Department of Health and Human Services PA 2008 guidelines, healthy American pregnant patients should exercise moderately for 150 min weekly spread throughout the week [3]. The PA recommendations during pregnancy for overweight and patients with obesity are not clear [76]. PA benefits in this population remain uncertain [101]. However, there is an expert consensus that pregnant patients with obesity could have long-term benefits for their children through PA before and/or during pregnancy [4].

## 5. Conclusions

The studies assessing the effectiveness of lifestyle interventions during pregnancy in optimizing GWG, and preventing GDM have declared conflicting results. Most studies support a preventive role of lifestyle interventions for extreme GWG. However, the results for GDM’s prevention are more heterogenous. Generally, exercise during pregnancy is safe, beneficial, and strongly recommended. Particularly, studies evaluating the effect of physical exercise during pregnancy, reported significant results in reducing excessive GWG, and preventing GDM. Future research is needed to explore the role of lifestyle interventions during pregnancy in restricting excessive GWG and preventing GDM.

## Figures and Tables

**Table 1:** Characteristics of pregnant women, interventions, and metabolic outcomes of included studies.

First Name Author, Publication Year	Population	Intervention	Control	Outcomes		
				Primary		Secondary
Tanentsapf, 2011 [33]	Normal-weight, overweight/obese	Diet or diet plus PA	Usual care	GWG		GDM
Oteng-Ntim, 2012 [36]	Overweight/obese	Diet and/or PA	Usual care	GWG		GDM
Thangaratinam, 2012 [32]	Normal-weight, overweight/obese	Diet and/or PA	Usual care	GWG		GDM
Yin, 2013 [55]	Low-risk, high-risk for GDM ^1^	PA	Usual care	GDM		N/a
Rogozińska, 2015 [56]	Low-risk, high-risk for GDM ^1^	Diet or diet plus PA	Usual care	GDM		Others ^2^
Russo, 2015 [44]	Low-risk, high-risk for GDM ^1^	PA	Usual care	GDM		N/a
Bain, 2015 [57]	Low-risk, high-risk for GDM ^1^	Diet and PA	Usual care	GDM		GWG
Sanabria-Martínez, 2015 [39]	Previous sedentary or low PA levels ^3^	PA	Usual care	GDM		GWG
Muktabhant, 2015 [58]	Normal-weight, overweight/obese	Diet and/or PA	Usual care	GWG		Others ^2^
Chen, 2015 [59]	Low-risk, high-risk for GDM ^1^	Fish oil supplementation	Usual care	GDM ^4^		Others ^2^
O’Brien, 2015 [8]	Normal-weight	Diet or diet plus PA	Usual care	GWG		GDM
Di Mascio, 2016 [60]	Normal-weight	PA	Usual care	Others ^2^		GDM
Ostadrahimi, 2016 [61]	Low-risk, high-risk for GDM ^1^	Fish oil supplementation	Usual care	GDM		Others ^2^
Song, 2016 [18]	Low-risk, high-risk for GDM ^1^	Diet or diet plus PA	Usual care	GDM		N/a
Magro-Malosso, 2016 [62]	Overweight/obese	PA	Usual care	GDM		N/a
Tieu, 2017 [63] ^5^	Normal-weight, overweight/obese	Diet	Usual care	GDM		Others ^2^
Zheng, 2017 [64]	Low-risk, high-risk for GDM ^1^	PA	Usual care	GDM		Others ^2^
Yu, 2017 [65]	Low-risk, high-risk for GDM ^1^	PA	Usual care	GDM		Others ^2^
i-WIP Collaborative Group, 2017 [66]	Normal-weight, overweight/obese	Diet and/or PA	Usual care	GWG		GDM
Shepherd, 2017 [67]	Low-risk, high-risk for GDM ^1^	Diet and PA	Usual care	GDM	GWG	Others ^2^
Bennett, 2018 [53]	Normal-weight, overweight/obese	Diet and/or PA	Usual care	GDM		N/a
Du, 2018 [19]	Overweight/obese	PA	Usual care	GWG	GDM	Others ^2^
Peaceman, 2018 [68]	Overweight/obese	Diet and PA	Usual care	GWG		GDM
Guo, 2018 [69]	Low-risk, high-risk for GDM ^1^	Diet and/or PA	Usual care	GDM		N/a
Davenport, 2018 [70]	Low-risk, high-risk for GDM ^1^	PA or diet plus PA	Usual care	GDM		N/a
Ming, 2018 [50]	Normal-weight	PA	Usual care	GDM		GWG
Nasiri-Amiri, 2019 [27]	Overweight/obese	PA	Usual care	GDM		N/a
Chatzakis, 2019 [71]	Overweight/obese	Metformin or vitamin D supplementation or probiotics supplementation or PA	Usual care	GDM		GWG
Doi, 2020 [72]	High-risk for GDM ^1^	PA	Usual care	GDM		N/a
Muhammad, 2020 [20]	Overweight/obese	PA	Usual care	GWG		GDM
Xing, 2020 [11]	Overweight/obese	PA	Usual care	GWG		GDM
Zhang, 2021 [10]	Low-risk, high-risk for GDM ^1^	Diet	Usual care	GDM		GWG
Davidson, 2021 [73]	Normal-weight, overweight/obese	Probiotics supplementation	Usual care	GDM		Others ^2^
Cantor, 2021 [74]	Normal-weight, overweight/obese	Diet and PA	Usual care	GDM		GWG
Díaz-Burrueco, 2021 [75]	Normal-weight, overweight/obese	PA	Usual care	GWG	GDM	N/a
Pascual-Morena, 2021 [76]	Overweight/obese	PA or metformin	Usual care	GDM	GWG	N/a
Teede, 2021 [77]	Normal-weight, overweight/obese	Diet and/or PA	Usual care	GWG		GDM
Tang, 2022 [78]	Low-risk, high-risk for GDM ^1^	Diet or probiotics supplementation or inositol supplementation or PA or diet plus PA	Usual care	GDM		N/a
Wu 2022, [45]	Overweight/obese	Diet and/or PA or metformin	Usual care	GDM		GWG
Wei, 2022 [16]	Low-risk, high-risk for GDM ^1^	Inositol supplementation	Usual care	GDM		Others ^2^
Tsironikos, 2022 [79]	High-risk for GDM ^1^	PA	Usual care	GDM		N/a
Tsironikos, 2023 [80]	High-risk for GDM ^1^	Diet and/or PA	Usual care	GDM		N/a

PA, physical activity; GWG, gestational weight gain; GDM, gestational diabetes mellitus; N/a, not applicable; i-WIP, International Weight Management in Pregnancy. ^1^ Pregnant women with any identified GDM risk factor; ^2^ Outcomes except for GWG or GDM; ^3^ PA less than 20 min, less than three times weekly; ^4^ Analysis for women only at low risk of pregnancy; ^5^ Analysis for dietary intervention arm compared to usual care.

**Table 2:** Effectiveness of lifestyle interventions on reducing gestational metabolic risk.

Dietary Interventions									
First Name Author, Publication Year	Population	Sample Size	Outcomes			Significance			
			Primary		Secondary	Primary Outcome		Secondary Outcome	
Thangaratinam, 2012 [32] ^1^	Normal-weight, overweight/obese	2560 for GWG; 409 for GDM	GWG		GDM	MD −3.84 kg, 95%CI −5.22, −2.45; *p*-value < 0.001		RR 0.39, 95%CI 0.23, 0.69; *p*-value 0.001	
Rogozińska, 2015 [56] ^1^	Low-risk, high-risk for GDM ^2^	1479	GDM		Others ^3^	RR 0.67, 95%CI 0.39, 1.15; *p*-value 0.15		-	
Muktabhant, 2015 [58] ^1^	Normal-weight, overweight/obese	835	GWG		Others ^3^	RR 0.77, 95%CI 0.66, 0.91; *p*-value < 0.001		-	
Song, 2016 [18] ^1^	Low-risk, high-risk for GDM ^2^	1455	GDM		N/a	RR 0.80, 95%CI 0.58, 1.10; *p*-value 0.1658		N/a	
Tieu, 2017 [63] ^4^	Normal-weight, overweight/obese	1279	GDM		Others ^3^	RR 0.60, 95%CI 0.35, 1.04; *p*-value 0.07		-	
i-WIP Collaborative Group, 2017 [66] ^1^	Normal-weight, overweight/obese	2018 for GWG; 1106 for GDM	GWG		GDM	MD −2.84 kg, 95%CI −4.77, −0.91; *p*-value n/a		OR 0.79, 95%CI 0.37, 1.69; *p*-value n/a	
Bennett, 2018 [53] ^1^	Normal-weight, overweight/obese	3388	GDM		N/a	RR 0.56, 95%CI 0.36, 0.87; *p*-value 0.009		N/a	
Guo, 2018 [69] ^1^	Low-risk, high-risk for GDM ^2^	2838	GDM		N/a	RR 0.75, 95%CI 0.60, 0.95; *p*-value n/a		N/a	
Zhang, 2021 [10]	Low-risk, high-risk for GDM ^2^	1848 for GDM; 1367 for GWG	GDM		GWG	OR 0.66, 95%CI 0.52, 0.82; *p*-value 0.0003		Std. MD −0.15, 95%CI −0.26, −0.05; *p*-value 0.004	
Teede, 2021 [77] ^1^	Normal-weight, overweight/obese	4938 for GWG; 3029 for GDM	GWG		GDM	MD −2.63 kg, 95%CI −3.87, −1.40; *p*-value n/a		OR 0.61, 95%CI 0.45, 0.82; *p*-value n/a	
Tang, 2022 [78] ^1^	Low-risk, high-risk for GDM ^2^	1421	GDM		N/a	OR 0.76, 95%CI 0.55, 1.05; *p*-value n/a		N/a	
Wu, 2022 [45] ^1^	Overweight/obese	1412 for GDM; 1015 for GWG	GDM		GWG	RR 1.10, 95%CI 0.83 1.46; *p*-value n/a		MD −1.95 kg, 95%CI −3.19, −0.71; *p*-value n/a	
Tsironikos, 2023 [80] ^1^	High-risk for GDM ^2^	3109	GDM		N/a	OR 0.73, 95%CI 0.51, 1.03; *p*-value 0.07		N/a	
**Supplementation Interventions**									
**First Name Author, Publication Year**	**Population**	**Sample Size**	**Outcomes**			**Significance**			
			**Primary**		**Secondary**	**Primary Outcome**		**Secondary Outcome**	
Chen, 2015 [59]	Low-risk, high-risk for GDM ^2^	4117	GDM ^5^		Others ^3^	Fish oil: RR 1.06, 95%CI 0.85, 1.32; *p*-value 0.60		-	
Ostadrahimi, 2016 [61]	Low-risk, high-risk for GDM ^2^	2455	GDM		Others ^3^	Fish oil: RR 0.97, 95%CI 0.74, 1.27; *p*-value n/a		-	
Chatzakis, 2019 [71] ^1^	Overweight/obese	154 for Vitamin D and GDM and GDM; 1061 for Probiotics and GDM; 841 for Probiotics and GWG	GDM		GWG	Vitamin D: RR 0.78, 95%CI 0.37, 1.62; *p*-value n/a	Probiotics: RR 1.08, 95%CI 0.78, 1.51; *p*-value n/a	Vitamin D supplementation: MD −0.4 kg, 95%CI −23.29, 22.49; *p*-value n/a	Probiotics supplementation: MD 0.5 kg, 95%CI −10.91, 11.92; *p*-value n/a
Davidson, 2021 [73]	Normal-weight, overweight/obese	1440	GDM		Others ^3^	RR 0.80, 95%CI 0.54, 1.20; *p*-value 0.28		-	
Tang, 2022 [78] ^1^	Low-risk, high-risk for GDM ^2^	2919	GDM		N/a	Probiotics: OR 0.57, 95%CI 0.34, 0.96; *p*-value n/a	Inositol: OR 0.82, 95%CI 0.43, 1.56; *p*-value n/a	N/a	
Wei, 2022 [16]	Low-risk, high-risk for GDM ^2^	1321	GDM		Others ^3^	RR 0.30, 95%CI 0.18, 0.49; *p*-value < 0.00001		-	
**Exercise Interventions**									
**First Name Author, Publication Year**	**Population**	**Sample Size**	**Outcomes**			**Significance**			
			**Primary**		**Secondary**	**Primary Outcome**		**Secondary Outcome**	
Thangaratinam, 2012 [32] ^1^	Normal-weight, overweight/obese	1057	GWG		N/a	MD −0.72 kg, 95%CI −1.20, −0.25; *p*-value 0.003		N/a	
Yin, 2013 [55]	Low-risk, high-risk for GDM ^2^	1089	GDM		N/a	RR 0.91, 95%CI 0.57, 1.44; *p*-value 0.68		N/a	
Russo, 2015 [44]	Low-risk, high-risk for GDM ^2^	3401	GDM		N/a	RR 0.72, 95%CI 0.58, 0.91; *p*-value 0.005		N/a	
Sanabria-Martínez, 2015 [39]	Previous sedentary or low PA levels ^6^	2631 for GDM; 2873 for GWG	GDM		GWG	RR 0.69, 95%CI 0.52, 0.91; *p*-value 0.009		MD −1.14 kg, 95%CI −1.50, −0.78; *p*-value < 0.001	
Muktabhant, 2015 [58] ^1^	Normal-weight, overweight/obese	603 for unsupervised exercise; 1298 for supervised exercise	GWG		Others ^3^	Unsupervised exercise: RR 0.83, 95%CI 0.71, 0.97; *p*-value 0.02	Supervised exercise: RR 0.75, 95%CI 0.63, 0.89; *p*-value < 0.001	-	
Di Mascio, 2016 [60]	Normal-weight	2059	Others ^3^		GDM	N/a		RR 0.41, 95%CI 0.24, 0.68; *p*-value n/a	
Song, 2016 [18] ^1^	Low-risk, high-risk for GDM ^2^	4512	GDM		N/a	RR 0.77, 95%CI 0.54, 1.09; *p*-value 0.1456		N/a	
Magro-Malosso, 2016 [62]	Overweight/obese	1502	GDM		N/a	RR 0.61, 95%CI 0.41, 0.90; *p*-value n/a		N/a	
Zheng, 2017 [64]	Low-risk, high-risk for GDM ^2^	1872	GDM		Others ^3^	Std. MD 0.62, 95%CI 0.43, 0.89; *p*-value 0.01		-	
Yu, 2017 [65]	Low-risk, high-risk for GDM ^2^	2164	GDM		Others ^3^	Std. MD 0.59, 95%CI 0.39, 0.88; *p*-value 0.01		-	
i-WIP Collaborative Group, 2017 [66] ^1^	Normal-weight, overweight/obese	7355 for GWG; 6755 for GDM	GWG		GDM	MD −0.72 kg, 95%CI −1.04, −0.41; *p*-value n/a		OR 0.66, 95%CI 0.53, 0.83; *p*-value n/a	
Bennett, 2018 [53] ^1^	Normal-weight, overweight/obese	2981	GDM		N/a	RR 0.62, 95%CI 0.50, 0.78; *p*-value < 0.001		N/a	
Du, 2018 [19]	Overweight/obese	1172 for GWG; 1120 for GDM	GWG	GDM	Others ^3^	MD −1.14 kg, 95%CI −1.67, −0.62; *p*-value < 0.0001	RR 0.71, 95%CI 0.57, 0.89; *p*-value = 0.004	-	
Guo, 2018 [69] ^1^	Low-risk, high-risk for GDM ^2^	5883	GDM		N/a	RR 0.70, 95%CI 0.59, 0.84; *p*-value n/a		N/a	
Davenport, 2018 [70] ^1^	Low-risk, high-risk for GDM ^2^	6934	GDM		N/a	OR 0.62, 95%CI 0.52, 0.75; *p*-value < 0.00001		N/a	
Ming, 2018 [50]	Normal-weight	2981 for GDM; 1688 for GWG	GDM		GWG	RR 0.58, 95%CI 0.37, 0.90; *p*-value 0.01 and RR 0.60, 95%CI 0.36, 0.98; *p*-value 0.04, respectively ^7^		MD −1.61 kg, 95%CI −1.99, −1.22; *p*-value 0.01	
Nasiri-Amiri, 2019 [27]	Overweight/obese	1441	GDM		N/a	RR 0.76, 95%CI 0.56, 1.03; *p*-value 0.07		N/a	
Chatzakis, 2019 [71] ^1^	Overweight/obese	902 for GDM; 1090 for GWG	GDM		GWG	RR 0.81, 95%CI 0.61, 1.06; *p*-value n/a		MD 0.96 kg, 95%CI −1.69, −0.23; *p*-value n/a	
Doi, 2020 [72]	High-risk for GDM ^2^	1467	GDM		N/a	RR 0.69, 95%CI 0.51, 0.94; *p*-value n/a		N/a	
Muhammad, 2020 [20]	Overweight/obese	745 for GWG; 665 for GDM	GWG		GDM	MD −0.88 kg, 95%CI −1.73, −0.03; *p*-value 0.04		RR 0.78, 95%CI 0.51, 1.19; *p*-value 0.25	
Xing, 2020 [11]	Overweight/obese	1405 for GWG; 1580 for GDM	GWG		GDM	Std. MD −0.21, 95%CI −0.32, −0.10; *p*-value < 0.001		RR 0.71, 95%CI 0.48, 1.04; *p*-value 0.081	
Pascual-Morena, 2021 [76] ^1^	Overweight/obese	1255 for GDM; 1159 for GWG	GDM	GWG	N/a	Aerobic		N/a	
						RR 0.51, 95%CI 0.26, 0.97; *p*-value n/a	MD −1.40 kg, 95%CI −3.47, 0.68; *p*-value n/a		
						Resistance			
						N/a	MD −1.35 kg, 95%CI −4.29, 1.59; *p*-value n/a		
						Combined			
						RR 0.75, 95%CI 0.47, 1.19; *p*-value n/a	MD −0.24 kg, 95%CI −1.68, 1.20; *p*-value n/a		
Teede, 2021 [77] ^1^	Normal-weight, overweight/obese	8714 for GWG; 7519 for GDM	GWG		GDM	MD −1.04 kg, 95%CI −1.33, −0.74; *p*-value n/a		OR 0.60, 95%CI 0.47, 0.75; *p*-value n/a	
Tang, 2022 [78] ^1^	Low-risk, high-risk for GDM ^2^	4830	GDM		N/a	OR 0.64, 95%CI 0.46, 0.88; *p*-value n/a		N/a	
Wu 2022, [45] ^1^	Overweight/obese	1110 for GDM; 351 for GWG	GDM		GWG	RR 0.821, 95%CI 0.60, 1.13; *p*-value n/a		MD −1.98 kg, 95%CI −3.50, −0.47; *p*-value n/a	
Tsironikos, 2022 [79]	High-risk for GDM ^2^	1508	GDM		N/a	OR 0.70, 95%CI 0.52, 0.93; *p*-value 0.02		N/a	
Tsironikos, 2023 [80] ^1^	High-risk for GDM ^2^	2742	GDM		N/a	OR 0.64, 95%CI 0.51, 0.80; *p*-value < 0.0001		N/a	
**Dietary Plus Exercise Interventions**									
**First Name Author, Publication Year**	**Population**	**Sample Size**	**Outcomes**			**Significance**			
			**Primary**		**Secondary**	**Primary Outcome**		**Secondary Outcome**	
Tanentsapf, 2011 [33]	Normal-weight, overweight/obese	1434 for GWG; 886 for GDM	GWG		GDM	MD −1.92, kg, 95%CI −3.65, −0.19; *p*-value 0.03		RR 0.74, 95%CI 0.52, 1.06; *p*-value n/a	
Oteng-Ntim, 2012 [36]	Overweight/obese	1228 for GWG; 1011 for GDM	GWG		GDM	MD −2.21 kg, 95%CI −2.86 −1.57 l; *p*-value < 0.00001		OR 0.80, 95%CI 0.58, 1.10; *p*-value 0.17	
Thangaratinam, 2012 [32] ^1^	Normal-weight, overweight/obese	1864 for GWG; 1233 for GDM	GWG		GDM	MD −1.06 kg, 95%CI −1.67, −0.46; *p*-value < 0.001		RR 1.18, 95%CI 0.78, 1.77; *p*-value 0.44	
Rogozińska, 2015 [56] ^1^	Low-risk, high-risk for GDM ^2^	4745	GDM		Others ^3^	RR 0.95, 95%CI 0.89, 1.22; *p*-value 0.65		-	
Bain, 2015 [57]	Low-risk, high-risk for GDM ^2^	3744 for GDM; 2707 for GWG	GDM		GWG	RR 0.92, 95%CI 0.68, 1.23; *p*-value 0.55		MD −0.76 kg, 95%CI −1.55, −0.03; *p*-value 0.06	
Muktabhant, 2015 [58] ^1^	Normal-weight, overweight/obese	3144 for diet and exercise counseling; 689 for diet and supervised exercise	GWG		Others ^4^	Diet and exercise counselling: RR 0.86, 95%CI 0.75, 0.98; *p*-value 0.02	Diet and supervised exercise: RR 0.71, 95%CI 0.59, 0.85; *p*-value < 0.001	-	
O’Brien, 2015 [8]	Normal-weight	446 for GWG; 243 for GDM	GWG		GDM	MD −1.25 kg, 95%CI −2.39, −0.11; *p*-value 0.03		RR 1.02, 95%CI 0.41, 2.57; *p*-value n/a	
Song, 2016 [18] ^1^	Low-risk, high-risk for GDM ^2^	6686	GDM		N/a	RR 0.85, 95%CI 0.70, 1.03; *p*-value 0.0922		N/a	
i-WIP Collaborative Group, 2017 [66] ^1^	Normal-weight, overweight/obese	8448 for GWG; 9342 for GDM	GWG		GDM	MD −1.00 kg, 95%CI −1.39, −0.61; *p*-value n/a		OR 0.88, 95%CI 0.72, 1.07; *p*-value n/a	
Shepherd, 2017 [67]	Low-risk, high-risk for GDM ^2^	6633 for GDM; 5052 for GWG	GDM	GWG	Others ^4^	RR 0.85, 95%CI 0.71, 1.01; *p*-value 0.07	MD −0.89 kg, 95%CI −1.39, −0.40; *p*-value < 0.001	-	
Bennett, 2018 [53] ^1^	Normal-weight, overweight/obese	7274	GDM		N/a	RR 0.90, 95%CI 0.77, 1.05; *p*-value 0.187		N/a	
Peaceman, 2018 [68]	Overweight/obese	1141 for GWG; 1007 for GDM	GWG		GDM	MD −1.58 kg, 95%CI −2.18, −0.99; *p*-value < 0.001		OR 0.92, 95%CI 0.61, 1.40; *p*-value 0.71	
Guo, 2018 [69] ^1^	Low-risk, high-risk for GDM ^2^	7024	GDM		N/a	RR 0.86, 95%CI 0.71, 1.04; *p*-value n/a		N/a	
Davenport, 2018 [70] ^1^	Low-risk, high-risk for GDM ^2^	7832	GDM		N/a	OR 0.90, 95%CI 0.74, 1.10; *p*-value 0.30		N/a	
Cantor, 2021 [74]	Normal-weight, overweight/obese	19752 for GDM; 20,090 for GWG	GDM		GWG	RR 0.87, 95%CI 0.79, 0.95; *p*-value n/a		MD −1.02 kg, 95%CI −1.30, −0.75; *p*-value n/a	
Díaz-Burrueco, 2021 [75]	Normal-weight, overweight/obese	3778 for GWG; 2675 for GDM	GWG	GDM	N/a	Std. MD −0.32, 95%CI −0.46, −0.17; *p*-value < 0.0001	OR 0.65, 95%CI 0.43, 0.98; *p*-value 0.04	N/a	
Teede, 2021 [77] ^1^	Normal-weight, overweight/obese	2942 for GWG; 3154 for GDM	GWG		GDM	MD −1.35 kg, 95%CI −1.95 −0.75; *p*-value n/a		OR 0.72, 95%CI 0.54, 0.96; *p*-value n/a	
Tang, 2022 [78] ^1^	Low-risk, high-risk for GDM ^2^	6315	GDM		N/a	OR 0.74, 95%CI 0.54, 1.01; *p*-value n/a		N/a	
Wu, 2022 [45] ^1^	Overweight/obese	6177 for GDM; 5102 for GWG	GDM		GWG	RR 0.90, 95%CI 0.74, 1.08; *p*-value n/a		MD −1.95 kg, 95%CI −3.19, −0.50; *p*-value n/a	
Tsironikos, 2023 [80] ^1^	High-risk for GDM ^2^	7673	GDM		N/a	OR 0.70, 95%CI 0.55, 0.90; *p*-value 0.005		N/a	

GWG, gestational weight gain; GDM, gestational diabetes mellitus; MD, mean difference; Kg, kilogram; CI, confidence interval; RR, risk ratio; N/a, not applicable; i-WIP, International Weight Management in Pregnancy; OR, odds ratio; Std., standard^1^. Studies with different components of lifestyle interventions; ^2^ Pregnant women with any identified GDM risk factor; ^3^ Outcomes except for GWG or GDM; ^4^ Analysis for dietary intervention arm compared to usual care; ^5^ Analysis for women only at low risk of pregnancy; ^6^ Exercise less than 20 min, less than three times weekly; ^7^ Based on different diagnosis criteria.

**Table 3:** Effectiveness of exercise interventions on reducing gestational metabolic risk.

Aerobic Exercise												
First Name Author, Publication Year	Population	Sample Size	Characteristics			Outcomes			Significance			
			Intensity	Duration	Supervision	Primary	Secondary		Primary Outcome		Secondary Outcome	
Di Mascio, 2016 [60]	Normal-weight	2059	Moderate	From the first or second trimester until the last trimester; three to four times weekly; 35–90 min per session	Supervised or not	Others ^2^	GDM		-		RR 0.41, 95%CI 0.24, 0.68; *p*-value n/a	
Magro-Malosso, 2016 [62]	Overweight/obese	1502	Moderate	From the first or second trimester until the last trimester or delivery; five to seven times weekly; 30–60 min per session	Supervised or not	GDM	N/a		RR 0.61, 95%CI 0.41, 0.90; *p*-value n/a		N/a	
Pascual-Morena, 2021 [76] ^1^	Overweight/obese	412 for GDM; 412 for GWG	Moderate	From the first or second trimester until the last trimester; three to five times weekly; 15–30 min per session	Supervised or not	GDM	GWG	N/a	RR 0.51, 95%CI 0.26, 0.97; *p*-value n/a	MD −1.40 kg, 95%CI −3.47, 0.68; *p*-value n/a	N/a	
**Resistance Exercise**												
**First Name Author, Publication Year**	**Population**	**Sample Size**	**Characteristics**			**Outcomes**			**Significance**			
			**Intensity**	**Duration**	**Supervision**	**Primary**	**Secondary**		**Primary Outcome**		**Secondary Outcome**	
Pascual-Morena, 2021 [76] ^1^	Overweight/obese	40	Light-to-moderate	From the second trimester until the last trimester; three times weekly; 35–40 min per session	Supervised	N/a	GWG	N/a	N/a	MD −1.35 kg, 95%CI −4.29, 1.59; *p*-value n/a	N/a	
**Aerobic Plus Resistance Exercise**												
**First Name Author, Publication Year**	**Population**	**Sample Size**	**Characteristics**			**Outcomes**			**Significance**			
			**Intensity**	**Duration**	**Supervision**	**Primary**	**Secondary**		**Primary Outcome**		**Secondary Outcome**	
Thangaratinam, 2012 [32] ^1^	Normal-weight, overweight/obese	1057	Moderate	From the first or second trimester until the last trimester or delivery; three times weekly; 30–60 min per session	Supervised or not	GWG	N/a		MD −0.72 kg, 95%CI −1.20, −0.25; *p*-value 0.003		N/a	
Yin, 2013 [55]	Low-risk, high-risk for GDM ^2^	1089	Light-to-moderate, moderate	From the first or second trimester until the last trimester; three to four times weekly; 30–60 min per session	Supervised or not	GDM	N/a		RR 0.91, 95%CI 0.57, 1.44; *p*-value 0.68		N/a	
Russo, 2015 [44]	Low-risk, high-risk for GDM ^2^	3401	Moderate	From the first or second trimester until the last trimester; one to three times weekly; 30–60 min per session	Supervised or not	GDM	N/a		RR 0.72, 95%CI 0.58, 0.91; *p*-value 0.005		N/a	
Sanabria-Martínez, 2015 [39]	Previous sedentary or low PA levels ^3^	2631 for GDM; 2873 for GWG	Light, light-to-moderate, moderate for GDM; very light, light, light-to-moderate-moderate for GWG	From the first or second trimester until the last trimester or delivery for both GDM and GWG; three to four times weekly for GDM, three to five times weekly for GWG; 45–60 min per session for GDM, 15–60 min per session for GWG	Supervised for GDM; supervised or not for GWG	GDM	GWG		RR 0.69, 95%CI 0.52, 0.91; *p*-value 0.009		MD −1.14 kg, 95%CI −1.50, −0.78; *p*-value < 0.001	
Muktabhant, 2015 [58] ^1^	Normal-weight, overweight/obese	603 for unsupervised exercise; 1298 for supervised exercise	Light-to-moderate, moderate	From the first or second trimester until the last trimester or delivery; two to every day weekly; 30–60 min per session	Supervised or not	GWG	Others ^4^		Unsupervised exercise: RR 0.83, 95%CI 0.71, 0.97; *p*-value 0.02		Supervised exercise: RR 0.75, 95%CI 0.63, 0.89; *p*-value < 0.001	-
Song, 2016 [18] ^1^	Low-risk, high-risk for GDM ^2^	4512	Light-to-moderate, moderate, moderate-to-high	From the first or second trimester until the last trimester or delivery; two to five times weekly; 30–60 min per session	Supervised or not	GDM	N/a		RR 0.77, 95%CI 0.54, 1.09; *p*-value 0.1456		N/a	
Zheng, 2017 [64]	Low-risk, high-risk for GDM ^2^	1872	Moderate	From the first or second trimester until the last trimester; three to most days weekly; 20–60 min per session	Supervised or not	GDM	Others ^4^		Std. MD 0.62, 95%CI 0.43, 0.89; *p*-value 0.01		-	
Yu, 2017 [65]	Low-risk, high-risk for GDM ^2^	2164	Moderate	From the first trimester until the last trimester; three to most days weekly; 20–60 min per session	Supervised or not	GDM	Others ^4^		Std. MD 0.59, 95%CI 0.39, 0.88; *p*-value 0.01		-	
i-WIP Collaborative Group, 2017 [66] ^1^	Normal-weight, overweight/obese	7355 for GWG; 6755 for GDM	Light-to-moderate, moderate	From the first or second trimester until the last trimester or delivery; two to three times weekly; 40–60 min per session	Supervised or not	GWG	GDM		MD −0.72 kg, 95%CI −1.04, −0.41; *p*-value n/a		OR 0.66, 95%CI 0.53, 0.83; *p*-value n/a	
Bennett, 2018 [53] ^1^	Normal-weight, overweight/obese	2981	Light-to-moderate, moderate	From the first or second trimester until the last trimester or delivery; two to three times weekly; 50–60 min per session	Supervised or not	GDM	N/a		RR 0.62, 95%CI 0.50, 0.78; *p*-value < 0.001		N/a	
Du, 2018 [19]	Overweight/obese	1172 for GWG; 1120 for GDM	Moderate	From the first or second trimester until the last trimester or delivery; two to most days weekly; 25–60 min per session	Supervised or not	GWG	GDM	Others ^4^	MD −1.14 kg, 95%CI −1.67, −0.62; *p*-value < 0.0001		RR 0.71, 95%CI 0.57, 0.89; *p*-value = 0.004	
Guo, 2018 [69] ^1^	Low-risk, high-risk for GDM ^2^	5883	Light-to-moderate, moderate	From the first or second trimester until the last trimester or delivery; two to five days weekly; 15–60 min per session	Supervised or not	GDM	N/a		RR 0.70, 95%CI 0.59, 0.84; *p*-value n/a		N/a	
Davenport, 2018 [70] ^1^	Low-risk, high-risk for GDM ^2^	6934	Light-to-moderate, moderate	From the first or second trimester until the last trimester or delivery; three to most days weekly; 15–60 min per session	Supervised or not	GDM	N/a		OR 0.62, 95%CI 0.52, 0.75; *p*-value < 0.00001		N/a	
Ming, 2018 [18]	Normal-weight	2981 for GDM; 1688 for GWG	Light-to-moderate, moderate	From the first or second trimester until the last trimester or delivery; one to three times weekly; 35–60 min per session	Supervised or not	GDM	GWG		RR 0.58, 95%CI 0.37, 0.90; *p*-value 0.01 and RR 0.60, 95%CI 0.36, 0.98; *p*-value 0.04, respectively ^5^		MD −1.61 kg, 95%CI −1.99, −1.22; *p*-value 0.01	
Nasiri-Amiri, 2019 [27]	Overweight/obese	1441	Light-to-moderate, moderate, moderate-to-high	From the first or second trimester until the last trimester or delivery or postpartum; two to everyday weekly; 15–60 min per session	Supervised or not	GDM	N/a		RR 0.76, 95%CI 0.56, 1.03; *p*-value 0.07		N/a	
Chatzakis, 2019 [71] ^1^	Overweight/obese	902 for GDM; 1090 for GWG	Light-to-moderate, moderate	From the first or second trimester until the last trimester or delivery or postpartum for both GDM and GWG; one to three times weekly for GDM, one to five times weekly for GWG; 30–60 min per session for GDM, 15–60 min per session for GWG	Supervised or not	GDM	GWG		RR 0.81, 95%CI 0.61, 1.06; *p*-value n/a		MD 0.96 kg, 95%CI −1.69, −0.23; *p*-value n/a	
Doi, 2020 [72]	High-risk for GDM ^2^	1467	Light-to-moderate	From the first or second trimester until the last trimester or delivery; two to three times weekly; 15–60 min per session	Supervised or not	GDM	N/a		RR 0.69, 95%CI 0.51, 0.94; *p*-value n/a		N/a	
Muhammad, 2020 [20]	Overweight/obese	745 for GWG; 665 for GDM	Moderate	From the first or second trimester until the last trimester or delivery; two to five times weekly; 15–60 min per session	Supervised or not	GWG	GDM		MD −0.88 kg, 95%CI −1.73, −0.03; *p*-value 0.04		RR 0.78, 95%CI 0.51,1.19; *p*-value 0.25	
Xing, 2020 [11]	Overweight/obese	1405 for GWG; 1580 for GDM	Moderate	From the first or second trimester until the last trimester or delivery; three to five times weekly; 25–60 min per session	Supervised or not	GWG	GDM		Std. MD −0.21, 95%CI −0.32, −0.10; *p*-value < 0.001		RR 0.71, 95%CI 0.48, 1.04; *p*-value 0.081	
Díaz-Burrueco, 2021 [75]	Normal-weight, overweight/obese	3778 for GWG; 2675 for GDM	Light-to-moderate	From the first or second trimester until the last trimester or postpartum; three to most days weekly; 15–60 min per session	Supervised or not	GWG	GDM	N/a	Std. MD −0.32, 95%CI −0.46, −0.17; *p*-value < 0.0001		OR 0.65, 95%CI 0.43, 0.98; *p*-value 0.04	N/a
Pascual-Morena, 2021 [76] ^1^	Overweight/obese	843 for GDM; 634 for GWG	Light-to-moderate, moderate	From the first or second trimester until the last trimester or delivery or postpartum; two to three days weekly; 40–60 min per session	Supervised	GDM	GWG	N/a	RR 0.75, 95%CI 0.47, 1.19; *p*-value n/a		MD −0.24 kg, 95%CI −1.68, 1.20; *p*-value n/a	N/a
Teede, 2021 [77] ^1^	Normal-weight, overweight/obese	8714 for GWG; 7519 for GDM	Light-to-moderate, moderate	From the first or second trimester until the last trimester or delivery or postpartum; three to most days weekly; 15–60 min per session	Supervised or not	GWG	GDM		MD −1.04 kg, 95%CI −1.33, −0.74; *p*-value n/a		OR 0.60, 95%CI 0.47, 0.75; *p*-value n/a	
Tang, 2022 [78] ^1^	Low-risk, high-risk for GDM ^2^	4830	Light-to-moderate, moderate	From the first or second trimester until the last trimester or delivery; two to five times weekly; 15–60 min per session	Supervised or not	GDM	N/a		OR 0.64, 95%CI 0.46, 0.88; *p*-value n/a		N/a	
Wu, 2022 [45] ^1^	Overweight/obese	1110 for GDM; 351 for GWG	Moderate	From the first or second trimester until the last trimester or delivery or postpartum; three times weekly; 30–60 min per session	Supervised	GDM	GWG		RR 0.821, 95%CI 0.60, 1.13; *p*-value n/a		MD −1.98 kg, 95%CI −3.50, −0.47; *p*-value n/a	
Tsironikos, 2022 [79]	High-risk for GDM ^2^	1508	Light-to-moderate, moderate	From the first or second trimester until the last trimester or delivery or postpartum; two to most days weekly; 15–60 min per session	Supervised or not	GDM	N/a		OR 0.70, 95%CI 0.52, 0.93; *p*-value 0.02		N/a	
Tsironikos, 2023 [80] ^1^	High-risk for GDM ^2^	2742	Light-to-moderate, moderate	From the first or second trimester until the last trimester or delivery or postpartum; three to most days weekly; 15–60 min per session	Supervised or not	GDM	N/a		OR 0.64, 95%CI 0.51, 0.80; *p*-value < 0.0001		N/a	

GDM, gestational diabetes mellitus; RR, risk ratio; CI, confidence interval; N/a, not applicable; GWG, gestational weight gain; MD, mean difference; Kg, kilogram; i-WIP, International Weight Management in Pregnancy; Std., standard; OR, odds ratio; Min, minute. ^1^ Studies with different components of lifestyle interventions; ^2^ Pregnant women with any identified GDM risk factor; ^3^ Exercise less than 20 min, less than three times weekly; ^4^ Outcomes except for GWG or GDM; ^5^ Based on different diagnosis criteria.

## Data Availability

Data are contained within the article.

## Supplementary Materials

The following supporting information can be downloaded at: https://www.mdpi.com/article/10.3390/jcm13123462/s1, Table S1: Search Strategy.
