# Supplementary material for: Gestational Metabolic Risk: A Narrative Review of Pregnancy-Related Complications and of the Effectiveness of Dietary, Exercise and Lifestyle Interventions during Pregnancy on Reducing Gestational Weight Gain and Preventing Gestational Diabetes Mellitus
Source: J Clin Med. 2024 Jun 13;13(12):3462. doi: 10.3390/jcm13123462 (PMC11204633; doi:10.3390/jcm13123462)
Supplement: Supplementary file 1 [file jcm-13-03462-s001.zip › jcm-2999693-supplementary.pdf]

Supplementary material

Table S1. Search Strategy.

PubMed	(diet OR nutrition) OR ((physical activity) OR (exercise)) AND ((obesity) OR (gestational weight gain) OR (gestational diabetes mellitus))<br><br>Filters applied: Meta-Analysis, Systematic Review, from 2000/1/1 – 2023/11/30
CDSR	((diet OR nutrition) OR ((“physical NEXT activit*”) OR exercise) AND (obesity) OR (gestational AND weight AND gain) OR (gestational AND diabetes AND mellitus))<br><br>with Cochrane Library publication date from Jan 2000 to Nov 2023, (Word variations have been searched)
Scopus	TITLE-ABS-KEY ((diet OR nutrition) OR ("physical activit*" OR exercise) AND (obesity) OR (gestational AND weight AND gain) OR (gestational AND diabetes AND mellitus)) AND (LIMIT-TO (DOCTYPE , "re")) AND (LIMIT-TO (EXACTKEYWORD , "Female") OR LIMIT-TO (EXACTKEYWORD , "Pregnancy")) AND (LIMIT-TO (LANGUAGE , "English")) AND (LIMIT-TO (PUBYEAR , 2023) OR LIMIT-TO (PUBYEAR , 2022) OR LIMIT-TO (PUBYEAR , 2021) OR LIMIT-TO (PUBYEAR , 2020) OR LIMIT-TO (PUBYEAR , 2019) OR LIMIT-TO (PUBYEAR , 2018) OR LIMIT-TO (PUBYEAR , 2017) OR LIMIT-TO (PUBYEAR , 2016) OR LIMIT-TO (PUBYEAR , 2015) OR LIMIT-TO (PUBYEAR , 2014) OR LIMIT-TO (PUBYEAR , 2013) OR LIMIT-TO (PUBYEAR , 2012) OR LIMIT-TO (PUBYEAR , 2011) OR LIMIT-TO (PUBYEAR , 2010) OR LIMIT-TO (PUBYEAR , 2009) OR LIMIT-TO (PUBYEAR , 2008) OR LIMIT-TO (PUBYEAR , 2007) OR LIMIT-TO (PUBYEAR , 2006) OR LIMIT-TO (PUBYEAR , 2005) OR LIMIT-TO (PUBYEAR , 2004) OR LIMIT-TO (PUBYEAR , 2003) OR LIMIT-TO (PUBYEAR , 2002) OR LIMIT-TO (PUBYEAR , 2001) OR LIMIT-TO (PUBYEAR , 2000))
